# A systematic scoping review of digital health technologies during COVID-19: a new normal in primary health care delivery

**DOI:** 10.1007/s12553-023-00725-7

**Published:** 2023-01-06

**Authors:** Costase Ndayishimiye, Henrique Lopes, John Middleton

**Affiliations:** 1 Association of Schools of Public Health in the European Region (ASPHER), 1150 Brussels, Belgium; 2 Comité mondial pour les apprentissages tout au long de la vie (CMAtlv), partenaire officiel de l’UNESCO, 75004 Paris, France; 3 Health Economics and Social Security Department, Jagiellonian University Medical College, 8 Skawińska, 31-066 Krakow, Poland

**Keywords:** primary health care, COVID-19, digital health, health technology, health care technology

## Abstract

This scoping review compiled information concerning digital health technologies (DHTs) evolution to support primary health care (PHC) during COVID-19 and lessons for the future of PHC. The identified literature was published during the COVID-19 peak years (2019–2021), retrieved from PubMed, Scopus, and Google Scholar, as well as hand searched on the internet. Predefined inclusion criteria were used, thematic analysis was applied, and reporting followed the PRISMA for Scoping Reviews.

A total of 46 studies were included in the final synthesis (40 articles, one book, two book chapters, one working paper, and two technical reports). These studies scrutinized various aspects of DHTs, entailing 19 types of DHTs with 20 areas of use that can be compressed into five bigger PHC functions: general PHC service delivery (teleconsultations, e-diagnosis, e-prescription, etc.); behavior promotion and digital health literacy (e.g., combating vaccine hesitancy); surveillance functions; vaccination and drugs; and enhancing system decision-making for proper follow-up of ongoing PHC interventions during COVID-19.

DHTs have the potential to solve some of the problems that have plagued us even prior to COVID-19. Therefore, this study uses a forward-looking viewpoint to further stimulate the use of evidence-based DHT, making it more inclusive, educative, and satisfying to people’s needs, both under normal conditions and during outbreaks. More research with narrowed research questions is needed, with a particular emphasis on quality assurance in the use of DHTs, technical aspects (standards for digital health tools, infrastructure, and platforms), and financial perspectives (payment for digital health services and adoption incentives).

**Supplementary Information:**

The online version contains supplementary material available at 10.1007/s12553-023-00725-7.

## Introduction

All over the world, the provision of fairer (equitable), comprehensive, and integrated health care is the aspiration that health care systems strive to achieve. It has been set out in the WHO and UNICEF-defined principle of primary health care (PHC) [1] *as* “*a whole-of-society approach to health that aims at ensuring the highest possible level of health and well-being and their equitable distribution by focusing on people’s needs and as early as possible along the continuum from health promotion and disease prevention to treatment, rehabilitation, and palliative care, and as close as feasible to people’s everyday environment*” (p2).

PHC principles were initially stated in the Alma-Ata Declaration of 1978 [2], which is regarded as a watershed moment in global health. 40 years later, in October 2018, global leaders gathered in Astana, Kazakhstan, for the Global Conference on Primary Health Care [3] to ratify the Astana Declaration [4] for proper PHC delivery. PHC is considered the most efficient and effective strategy for achieving universal health because it focuses on how to effectively deliver health care and services to everyone, everywhere. PHC, on the other hand, differs from the primary care concept, which is often used interchangeably. The shorter term, primary care (PC), refers to a more limited set of “family doctor-type” services provided to individuals [5].

The response to the Sars-Cov-2 pandemic, also known as COVID-19 [6], revealed missed opportunities to provide PHC to all. Many health-care services have been postponed in many countries in order to focus on COVID-19 patients [7–9]. Other reports show how health systems have faced compounding weaknesses in delivering PHC during COVID-19 [10], including underfunding and postponing some care services.

Nonetheless, in outbreaks like COVID-19, PHC delivery is hampered by several obstacles that must be overcome. While there may be other ways to improve PHC delivery during epidemics and pandemics, digital health technologies (DHTs) would be among the potential strategies. Thus, understanding how digital health has adapted to PHC delivery during the 2019 coronavirus pandemic around the world is particularly important.

## Study context

The WHO defines digital health as a broad umbrella term including eHealth (including mobile health) as well as new disciplines such as [the] application of sophisticated computing sciences in “big data,“ genomics, and artificial intelligence [11]. For this scoping review, themes were created in reference to the WHO classification of digital health interventions [12]: “client consultations and health information dissemination, citizen-based reporting (mobile apps), on-demand information (national hotlines), and telemedicine (telehealth).” Virtual models of PHC, on the other hand, implement the WHO framework for integrated people-centred health services (IPCHS) by integrating care at the primary-secondary interface (e.g., between general practitioners (GPs) and specialists), integrating health and social care (e.g., between primary care, allied health, and social services), and orienting care provision around people’s needs (i.e., people-centred) [13].

Previous studies concentrated exclusively on short-term adjustments to deliver PHC via DHTs and on the early days to months of the COVID-19 outbreak, resulting in less coverage of the literature. Our study, on the other hand, is new in the sense that it covers the entire pandemic’s peak years (i.e., 2019 to 2021) and aims to stimulate long-term thinking about how to make DHT more inclusive, educative, and satisfying to people’s needs, whether under normal conditions or during outbreaks. It also covers the necessity for long-term planning, considering new difficulties that arose as a result of the use of DHTs during the COVID-19 crisis, such as hospital hacking and other unlawful cyber-attacks on medical centres and medical technologies.

## Materials and methods

The review was performed in accordance with the Arksey and O´Malley scoping review methodology [14], which involves five mandatory steps, namely: (1) defining research questions; (2) identifying relevant literature; (3) selecting publications; (4) data extraction; (5) data analysis, summary, and result reporting; and optional step 6, which is consultations. The Preferred Reporting Items for Systematic Reviews and Meta-Analyses for Scoping Reviews (PRISMA-ScR) checklist was used [15].

### Defining resarch questions

The central review question (RQ) was “how has digital health technology (DHT) evolved to provide PHC in the midst of the COVID-19 pandemic?“ To better answer this central question, we also derived two secondary questions (RQ1 and RQ2). The first (RQ1) was about PHC’s response and adaptation to COVID-19 with a focus on DHTs:

- The types of technologies used for delivering PHC during COVID-19.

The second (RQ2) focused on what DHTs were used for:

- The purposes for which DHTs were used; the interventions using digital technologies; the importance of using these digital methods during COVID-19.

### Identification of studies

Studies were identified by searching the Medline database via PubMed, Scopus, and Google Scholar. Key terms were derived from three major concepts: “primary health care”, “digital health” and “COVID-19”. And then, for each of these three concepts, related keywords were used for searching (in MeSH terms where applicable), in titles, and in abstracts (see the supplementary material, **Table S1**). Additionally, a broad internet hand search for pertinent grey literature and a manual scan of the reference list of the studies that were included were conducted.

### Selection and handling of studies

The retrieved records were deduplicated via Mendeley reference manager. The study selection process involved two steps: abstract screening and full-text evaluation based on predetermined inclusion and exclusion criteria (Table 1). Two researchers working independently screened the studies and reached an agreement rate of 97% (i.e., greater than the 80% suggested by researchers like Lavac et al. [16]). Thus, there was no need to appoint a third independent researcher to make the final decision on which studies to include.

**Table 1:** Inclusion and exclusion criteria for studies

Inclusion criteria	Exclusion criteria
♣ Studies describingDHTs to provide PHC during the COVID-19 pandemic ♣ Peer-reviewed empirical studies, technical reports, working papers, and books/chapters ♣ Full text publications ♣ Publications in English language ♣ Dates limits: December 2019 to December 2021	♣ Studies on DHTs without a focus on the COVID-19 pandemic ♣ Studies with a lack of focus on PHC ♣ Conference abstracts, editorials, communications, commentary, etc. ♣ Other languages

### Data extraction and charting

The Excel spreadsheets were specifically designed in accordance with RQs 1–2, allowing for the synthesis of important themes and, where appropriate, assigning codes to aid in subsequent analysis (see Tables 2 and 3 in the section of results).

### Study analysis, summarising, and reporting of results

Generally, we followed three steps suggested by Levac et al. [16] to uniformize scoping investigations: first, to analyse the data; second, to report the results; and third, to apply the meaning to the findings. Specifically, however, we incorporated a six-step iterative process for thematic content analysis and synthesis: (i) getting to know the data; (ii) assigning a preliminary code; (iii) searching for themes; (iv) reviewing themes; (v) finalising themes; and (vi) generation of a report. The overall results were presented following the PRISMA-ScR, while the final report included a synthesis of themes in tables tailored to answer the RQs stated in step one.

### Consultations

The purpose of including consultations in this study, as is consistent with other scoping review researchers [16], was to ensure obtaining additional sources of information, viewpoints, meaning, and relevance to the scope of the research. Preliminary results were discussed with experts in PHC. Most of the experts consulted are members of organisations providing responses to the COVID-19 crisis, such as the Association of Schools of Public Health in the European Region (ASPHER), the WHO Regional Office for Europe, the COVID-19 Health System Response Monitor, and the European Observatory on Health Systems and Policies.

## Study results

### Overview of the included studies

**Fig. 1:**
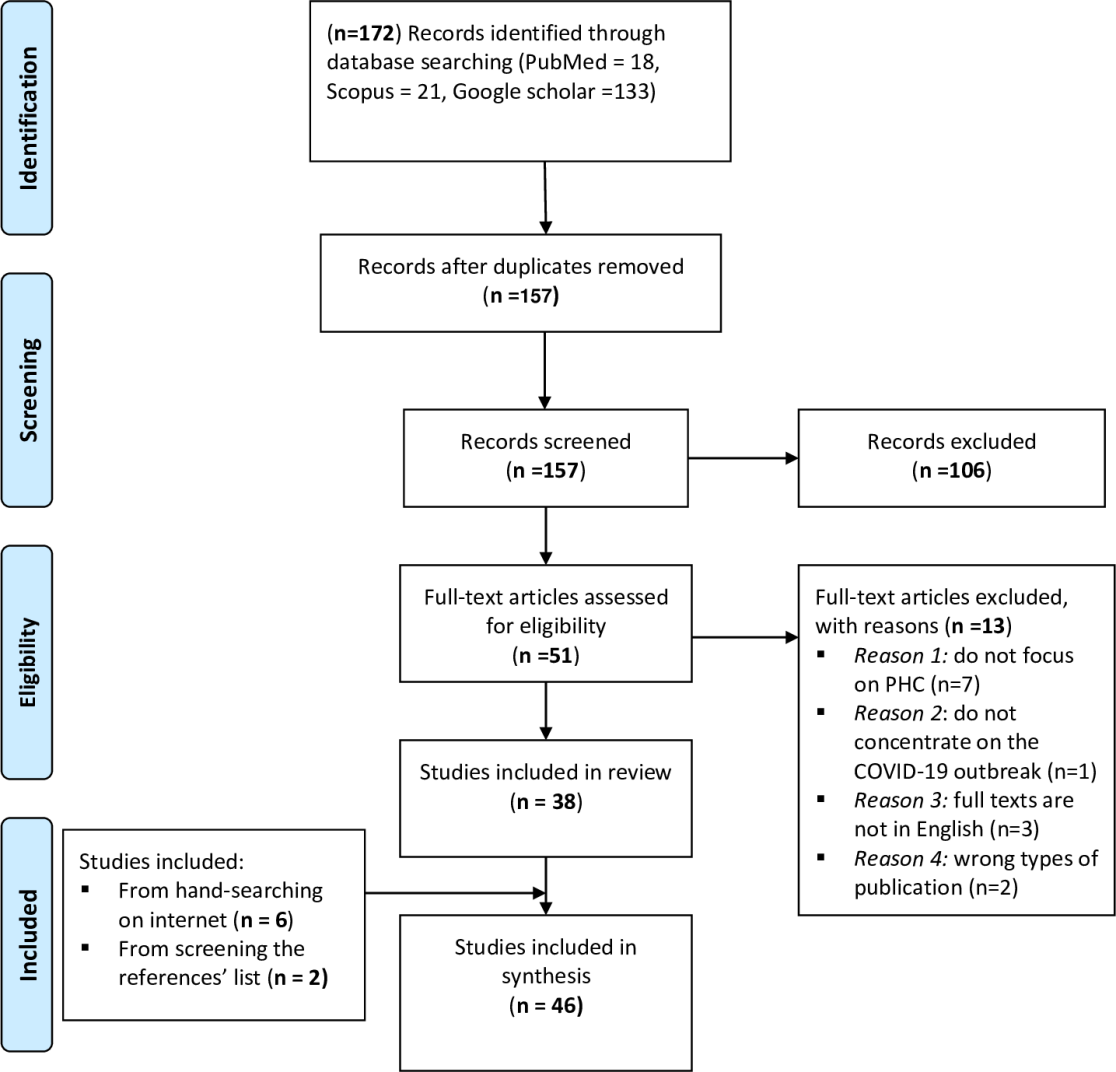
PRISMA Flowchart of the Results

The database search yielded 172 records (PubMed = 18, Scopus = 21, and Google Scholar = 133). After removing 15 duplicates, a total of 157 articles were screened by title and abstract, with 106 being subsequently excluded. The full-text versions of 51 records were evaluated for eligibility, with 13 eventually excluded. Finally, the reference lists of the 38 full-text studies that met the inclusion requirements were checked. Additional relevant records (n = 2) were identified from manual scanning of reference lists and (n = 6) from hand-searching on the internet. As a result, the final synthesis included a total of 46 records (Fig. 1), with 40 being journal articles [13, 17–55], and six being grey literature [56–61]. The six documents added from manual internet searches included a book [56] about why health tech started to transform public health during COVID-19, with key illustrations of changes in PHC thanks to telemedicine and AI, wearables, social media, and digital therapeutics; two book chapters. [57, 58] about DHTs helping a rapid shift in healthcare services due to the COVID-19 pandemic; one working paper [61] about empowering the PHC workforce to make the most of the digital revolution during COVID-19; and two reports [59, 60] about PHC and the rise of DHTs during the COVID-19 pandemic.

### Thematic synthesis to respond to study questions

To respond to RQ1, major classes of themes (n = 19) were recorded (Table 2). The top two of these 19 categories included the eHealth category, which comprised things like telehealth, e-prescriptions, and electronic health records; and the mHealth category, which included many subcategories such as apps on personal phones and mobile electronic records linking patients with PHC professionals. Generally, e-prescription, e-referral, digital sensors, wearables, e-mental health, telepsychiatry, telemedical stethoscopes, telemedical ECGs, telemonitoring, and teleconsultation, among other DHTs, were extensively adopted in response to the coronavirus pandemic. Additionally, big data and other DHTs, such as check-in kiosks, emails, online patient portals, self-management home monitoring devices, and chatbots, were also noted.

**Table 2:** Thematic synthesis of reviewed literature

RQ1—PHC response and adaption to COVID-19 with a focus on DHTs: • The types of technologies used for delivering PHC	
Key themes	References
***mHealth***: App-based telemedicine, mobile telemedicine, call centers, telephone-based care, SMS appointment systems, call helplines, mobile electronic patient records, mHealth for medication adherence, mHealth for health surveys, mHealth for surveillance and monitoring, mHealth for vital sign checks, national hotlines	(13,17–24,28,39,50,52–55,57,62)
***eHealth***: telehealth, telemedecine, e-prescription, e-referral, e-Mental health, telepsychiatry, clinic-based telehealth, telehealth visits	(20–27,29,51,54,57)
***Devices***: wearables, wearable health devices, portable electrocardiograms, telemedical stethoscopes, telemedical ECGs, remote monitoring devices, laboratory-on-a-chip’ devices	(28,29,39,50–55,62)
***Digital tracing***	(30,31)
***Testing, self-testing (self-screening)***	(32,33)
***Websites, Platforms, Trials Platform***	(27,34–38)
***Monitoring***: telemonitoring; home monitoring, remote monitoring, remote patient monitoring, remote monitoring systems, online monitoring	(13,18,19,21,28,38–41,50–52)
***TeleConnect***	(42)
***Digital health literacy technologies***	(43–45)
***Consultations***: tele-consultation, virtual consultations, video consultations	(13,17–19,28,29,39,40,45,50,51,53–55,62)
***Sensing***: medical sensor, digital sensor	(28,41,51,53–55,62)
***Internet of Things (IoT)***	(41,57)
***Artificial intelligence (AI)***	(28,39,50–55,62)
***Big data***	(41,57)
***Geospatial technology***: geospatial reporting systems, geospatial moinitoring, geospatial patient location, trends analysis.	(46)
***Others***: check-in kiosks, emails, online patient portals, self-management home monitoring devices, chatbots	(28,51,53–55,62)

To respond to RQ2 about what DHTs were used for in PHC during the COVID-19 pandemic, (n = 20) key areas of use were recorded, which were eventually classified into five big areas (Table 3). Firstly, DHTs were used to support the provision of general PHC services. This included using DHTs to practise general care (teleconsultations, diagnosis, testing, etc.), to manage patients with chronic conditions, mental health care, telehealth visiting options, and to digitally link physicians and bring the healthcare system closer together. Secondly, DHTs were used for communicating, informing, and educating (literacy) roles. This included behaviour change promotion (e.g., to combat vaccine hesitancy) and digital health literacy. Thirdly, DHTs were used for monitoring and surveillance purposes, such as monitoring patients’ symptoms and disease diffusion. Fourth, DHTs were used for vaccination and drug issues, such as e-prescription and control of undesired effects. The fifth use of DHTs was to enhance system decision-making for proper follow-up of ongoing PHC interventions and policy change when needed.

**Table 3:** Thematic synthesis _ RQ2 RQ2—What DHTs were used for during the COVID-19 pandemic?

References	Main areas	Subareas
(13,17–19,23,25–30,32,33,38–42,45,47,50,51,53–55,57,62)	***SUPPORTING PROVISION OF HEALTH SERVICES***	1. general practice for care provision (teleconsultations, diagnosis, testing, etc.)
		2. chronic disease management
		3. mental health illnesses management (e.g., telepsychriatry)
		4. palliative care
		5. telehealth visits 6. digitally connecting physicians across the health care sytem
		7. digitally mediated delegation in team-based primary care
		8. employing AI for infection detection and treatment
(24,34,43–45,48,62)	***COMMUNICATING, INFORMING, AND EDUCATING (LITERACY)***	9. communicating information on COVID-19
		10. behavior change promotion and digital health literacy (infection control, limiting inaccurate information spread, combating vaccine hesitancy, etc.)
(13,18,19,21,27,28,30,31,34–41,50–52)	***MONITORING AND SURVEILLANCE***	11. patients’ monitoring and surveillance
		12. symptoms monitoring
		13. controlling disease diffusion
		14. tracing
		15. enforcing isolation, self-isolation, and quarantining
(13,18,36–41,50–52,19,21,27,28,30,31,34,35) (28,45,49,51,53–55,62)	***VACCINATION AND DRUGS***	16. vaccine roll-out
		17. e-prescriptions
		18. controlling adverse reactions
(28,45,49,51,53–55,62) (38,52)	***PHC SYSTEM DECISION-MAKING***	19. tracking the impact of interventions
		20. policy decisions

## Discussion

This study compiled information concerning how DHTs evolved to support the PHC sector during COVID-19 and lessons for the future improvement of PHC. A total of 46 relevant studies were included in the final synthesis. These studies scrutinised various aspects of DHTs, entailing 19 types of DHTs with 20 areas of use that can be compressed into five bigger functions: general PHC service delivery (teleconsultations, diagnosis, testing, etc.); behaviour promotion through information dissemination and digital health literacy; surveillance functions; vaccination and drug issues; and enhancing system decision-making for proper follow-up of ongoing PHC interventions during COVID-19. The identified literature has been published during the COVID-19 peak years (2019–2021), and many of the included articles cover DHTs to ensure continued PHC delivery during COVID-19, health promotion, and information. These are important to the section that follows, with a forward-looking discussion of the future implications of DHTs in PHC.

### Context of the COVID-19 pandemic

#### DHTs to ensure continued PHC delivery during COVID-19

PHC practise has been impacted by the COVID-19 outbreak in many dimensions. DHTs, on the other hand, have evolved and adapted as a result. Telemonitoring, apps, wearable devices, and artificial intelligence are all examples of this. Previous studies have claimed that digital technology acceptance and implementation in PHC have been reluctant and delayed [63–65]. However, COVID-19 appears to have opened new doors and accelerated adoption rates. Primary care providers (PHCPs) depended heavily on DHTs to continue providing care during the COVID-19 epidemic. We found that DHTs allowed them to provide a wide variety of services, from diagnosis to treatment and palliative care, as well as preventative and vaccine drives. In addition, DHTs were utilised to increase the number of non-COVID-19 consultations and referrals to secondary care [9, 66] that would have been otherwise dismissed due to COVID-19’s early responses, such as cancelling non-urgent treatment, implementing lockdowns, and so on.

Due to the huge demand for other COVID-19 commitments, PHC centres were possibly understaffed. This would require different and inventive solutions in order to ensure that patients received sufficient care. Research shows that DHT use in primary (and secondary) care has risen at previously unheard-of rates since then [67, 68]. In the early days of the pandemic, an increase in the use of remote consultations in PHC was extensively noted. In April 2020, for example, 64–80% of PHCPs in the United Kingdom and the Netherlands used video consultations, 13% used telemonitoring, and 73% used electronic asynchronous consultations [69]. The use of DHTs in hospitals appears to be increasing significantly. At one academic hospital in New York, for example, the use of video consultations has increased by 8729% [70]. Remote monitoring apps for suspected or confirmed COVID-19 patients have equally evolved in hospitals, in addition to video consultations. The COVID-19 box is an example of a home monitoring system that allows patients to check their vital signs three times a day and have daily video chats with the hospital [68]. In family medicine, the remote follow-up of patients at elevated risk of worsening with moderate or severe COVID-19 has brought added value. In this case, unnecessary visits to the emergency room were avoided, and recovery at home was facilitated instead. In remote patient management programs, systems incorporating the collaboration of primary and secondary health care professionals (TeleConnect, for example) have evolved [71]. This has a lot of potential because it connects not only patients and care providers but also care providers among themselves. These technologies either connect PCPs between themselves or with specialists, a habit that was being done in person before the COVID-19 pandemic. These vital connections are therefore critical to patient care by facilitating care coordination and improving the patient’s managed care. Furthermore, it could allow patients to stay at home longer while still receiving all the care they require.

Digital sensors were among the other DHTs that were used for a variety of applications [41], including chronic condition management, diagnosis, and testing, among others. Many examples exist that show how sensors developed during the COVID-19 era can revolutionise how current and future PHC care is delivered. For example, a sensor created at Johns Hopkins University [72] can revolutionise virus diagnostics (i.e., virus testing). During early testing, the sensor detected SARS-CoV-2 in saliva samples with 92% accuracy, which was comparable to PCR assays. The sensor was also extremely effective at detecting the presence of other viruses, such as H1N1 and Zika. Researchers have also demonstrated that this sensor, which requires no sample preparation and little operator skill, has a strong advantage over conventional testing methods, particularly for population-wide testing. It has been shown to overcome the limitations of the two most common forms of COVID-19 tests, namely the PCR and rapid tests. Its key novelty is that it is a label-free technology, which means it does not require any additional chemical modifications such as molecular labelling or antibody functionalization. The latter implies that this sensor could be used in wearable devices in the future.

#### DHTs to ensure health literacy, promotion, and quality during COVID-19

Digital health literacy is another key aspect of DHT use that has been observed in the COVID-19 pandemic. In Pakistan, for example, it has helped improve maternal, child, and family health in primary healthcare settings in disadvantaged areas [43]. It has also aided in improving patient safety and directing patients to other health and social services. Community healthcare workers (CHWs) were also valuable in this process by using smartphones and internet-based digital health literacy initiatives to empower disadvantaged women. Research has shown that literacy and awareness-raising interventions in PHC settings are more beneficial to underserved women in other areas of maternal and child health, such as nutrition and food security, chronic disease management and multimorbidity, and mental health counselling [43]. Moreover, literacy in digital health interventions for patients can, for example, provide skills in short message systems (SMS) for cardiac patients, skills in mobile services for patients with diabetes, and skills in mobile devices that allow patients to access online consultations from a physician. Other skills that could be improved include interventions that empower patients to provide better information needed by healthcare providers and better record-keeping and management of patient information.

From a clinical and public health perspective, digital health literacy during COVID-19 shares exactly two wider visions with regular health literacy, across the three domains of health: health care, disease prevention, and health promotion. These are individual skills regarding health information. That is, information that enhances preferred actions in the care relationship between a “patient” and their “caregivers”. This, therefore, defines a clinical vision. Furthermore, this encompasses a broader set of cognitive, social, and civic skills. These skills enable a patient to use the information and public services to gain greater control over his or her own condition(s) (i.e., a patient gains understanding of his or her illness) and, finally, to make decisions to solve community health problems (i.e., a patient participates in collaborative efforts to find solutions to community health issues). The latter defines a public health vision that is considered a key element in decision-making, health promotion, and preventing ill-health. Importantly, patients access, understand, evaluate, and apply skills related to using health information. This illustrates exactly the skills aimed at strengthening the promotion of self-care and self-management of one’s health. All of these are very important because the individuals who have acquired them can use the information to improve their health. S/he would understand, for example, why it is important to use a face mask for COVID-19, to maintain social distance, to take medications as prescribed by a healthcare provider, and so on. This would reduce the gap in information between the patient and her/his care provider (i.e., reduce information asymmetry), which would help to better manage the patient’s health. In other words, it will enhance informational inclusion from the patient’s perspective. Therefore, the patient could, for example, determine, on the basis of the packaging of a pharmaceutical product, how often this product should be taken per day. S/he would also be able to understand what her/his doctor says, and s/he would be able to find information about her/his disease. In this case, the patient would become a good health care partner. To reiterate, improving digital health literacy would not only be a potential solution for successfully embracing digital technologies in healthcare, but it would also allow patients to be aware of and own their health conditions, allowing for better partnerships in delivering health care for both individuals and society as a whole.

Overall, studies show that DHTs were widely used during the COVID-19 pandemic, and they showed the potential to improve care (including health-care quality) [73–76]. Their promising applications are still being explored. Thus, we need to make sure we do not miss out on the chance provided by COVID-19 to apply DHTs in our daily PHC practice.

#### New DHTs concerns during COVID-19

Despite the scope of our study not being focused on the limitations of DHs or why the healthcare sector has been reluctant to accelerate the use of DHTs in the past, research claims that healthcare has been slow to adopt digital technologies while their proliferation has revolutionised many other industries by increasing efficiency and effectiveness [63]. Healthcare organizations, particularly physician practices, have been noticeably slow to adopt such technologies, voicing some challenges. Other researchers have generally looked at potential barriers to DHT use in the past [77–80], especially telehealth [81], but new specific negative concerns have emerged during COVID-19. To mention a few novel issues (only the more immediate and illegal aspects): (i) hackers attacking hospitals, clinical analysis centres and medical cyber centres for extorting money, capturing patient data, and preventing clinical activity; (ii) blackmailing people due to clinical data disclosure; and (iii) the threat of introducing the virus into electronic medical devices. We recommend conducting thorough research on the new negative effects of DHTs that appeared in the COVID-19 era.

### A forward look

Despite the unwelcome trigger, the COVID-19 pandemic has influenced how DHTs are perceived and used in the PHC community. It has led PHC providers and hospitals to use DHTs more extensively. Although there is no one-size-fits-all answer to the challenges facing PHC nowadays, the application of DHTs is one potential solution. We believe it is necessary to further stimulate the application of DHT with evidence, making it inclusive, educative, and more satisfying to people’s needs, whether in normal circumstances or during outbreaks. We, therefore, suggest the following considerations to improve the delivery of PHC in the future:

- If we have to grasp the DHT opportunity in healthcare properly, digital health literacy should be reinforced as it can be a potential barrier for both providers and users (patients).
- Previous studies show vulnerable groups of people [45], such as immigrants, the elderly, and women, as well as racial and ethnic disparities [82]; they should be tactically targeted due to their increased risks of digital exclusion (mainly due to worsening gaps in digital health literacy, along with other compounding issues, including language, financial, and difficulties in social acceptance and integration).
- As highlighted throughout this study, DHTs (e.g., sensors and wearables) are already being used in multiple scenarios related to chronic disease management at the PHC level [41]. As a result, their potential benefits should be continued after the pandemic has passed, because chronic diseases have been, are, and will undoubtedly continue to be a burden on healthcare systems. In this regard, DHTs may alleviate pre-existing pressure as a result of the latter.
- The PHC context integrates multiple disciplines into cohesive teams. The COVID-19 pandemic has prompted or accelerated the use of remote patient management programs, such as TeleConnect [42], which allow collaboration (e.g., between primary and secondary health care professionals). These technologies would make it easier for PCPs to work together or with specialists without having to rely exclusively on in-person collaboration. As such, this could potentially allow patients to be treated at home while still getting nearly all the care they need.
- (To this end,) these vital connections between various levels of care provision would be essential for providing coordinated care for patients, facilitating transitions between care settings, and improving the patient’s experience with their managed care.
- With the reduced number of in-person visits during the pandemic, virtual services enhanced by DHTs, such as remote monitoring [41, 52], have shown significant value. To proactively adapt and expand telemonitoring programs in the changing conditions of an increasingly virtualized healthcare system, they should revisit the scope and expectations of telehealth interventions, streamline virtual patient onboarding processes, and personalise patient information collection to build stronger virtual relationships and a more comprehensive assessment of patient safety.
- Some novel care models have evolved to address innovative solutions to tackle medical undersupply because of a shortage of human resources in healthcare (medical professionals, for example), as well as demographic and structural constraints. Medical delegation and team-based DHTs [29] appear to be a potential strategy to address these issues; however, this will require strong but smoother PHC communication structures and mechanisms to reassure that patients and caregivers are receiving a delegated task for a therapy-related duty. Thus, these evolutionary (if not revolutionary) processes could be made more productive and efficient by using DHTs.
- Should the big data generated by DHTs be used to improve future PHC research and development to deliver enhanced patient care, then decentralized and specialized professionally led primary care research centers (and possibly research networks) should be established to stay in the loop of medical research ethics and deontologies. Although it appears to be automatically stored in health care system repositories, this type of data requires extra protection [83, 84] and hence cannot be gathered and used routinely. Specialized research entities (in addition to the current research clearance bodies) will be needed at multiple levels, from the national level to the local level.
- Finally, before DHTs are fully integrated into PHC, a significant amount of research will be required, and they should be designed to suit their function—improving a patient’s care rather than exacerbating the problem. Teleconsultation (or telemonitoring) for a patient with psychiatric issues, for example, might not be implemented in the same way as it should be for patients in internal medicine. Telepsychiatry would almost certainly be feasible and acceptable for some people (perhaps those with minor issues), but would probably impair the outcomes for others (those patients with advanced mental health problems, for example).

## Implications for future research

Our research found that DHTs have potential in the current and future PHC industry, whether in health emergencies or in normal conditions. A few more things need further studies with narrower research questions. Firstly, there is a lack of clarity around the legal framework and licensing requirements for digital health tools. The second is about quality issues with the use of digital health, skills, and training. The third area of focus is the technical aspects of digital health tools, such as standards for interoperability, infrastructure, and platforms. Last but not least, financial considerations, such as payment for digital health services and incentives for adoption.

## Study limitation

Scoping reviews have some drawbacks as a research methodology. For instance, the included studies’ quality assessment is not as explicit [16]. Additionally, our study used broad RQs to cover more ground, which resulted in only a superficial examination of several facts. Therefore, additional in-depth research on specific research topics is required. Furthermore, we restricted the study period to the peak years of the COVID-19 pandemic (i.e., 2019 to 2021), and we only considered studies that were written in English. Despite the aforementioned limitations, we believe this study offers structured and helpful information on how DHTs evolved to support the PHC sector during COVID-19 and lessons for the future improvement of PHC.

## Conclusion

DHTs during COVID-19 were essential for PHC delivery. In this context, DHTs were commonly used for a variety of purposes, including providing virtual care services, providing clinical support, monitoring the quality of care, mapping and monitoring the spread of the coronavirus, as well as tracking supplies of drugs and vaccines. These DHTs have already opened up a wealth of possibilities and opportunities for shaping the future of PHC. Their incorporation into clinical support tools and referral systems can aid in care coordination and continuity across primary, secondary, acute, and long-term care servicesaged care services. They can help to prevent duplicate care processes and improve communication between PCH providers themselves and between patients. Additionally, they can help to avoid unplanned hospitalizations and visits to hospitals. DHT’s power to catalyse change is significant, so it is important that people are well-educated about digital health (digital health literacy), policy and practice are enhanced, and communication is effective. Some DHTs also provide the potential to address some of the shortages in the PHC workforce that are facing health systems internationally. For this to be a reality, there will be a need for cultural change in health care, as well as an investment in training and in new technology. Health systems should be committed to the goals of PHC and its values, and thus DHT inclusiveness and equity should be ensured to keep up with the changes that are necessary to provide care for all. Furthermore, there should be appropriate caution when using DHT approaches to ensure that the benefits are realized and any potential harms are avoided

## Electronic supplementary material

Below is the link to the electronic supplementary material.

Supplementary Material 1

## Data Availability

Not applicable.

## List of abbreviations

DHT: Digital Health Technology
PHC: Primary Health Care
PCP: Primary CarePphysician
GP: General practitioner
IPCHS: Integrated People-Centred Health Services
UHC: Universal Healthcare Coverage
RQ: Research Question
PRISMA-ScR: Preferred Reporting Items for Systematic Reviews and Meta-Analyses for Scoping Review
ASPHER: Association of Schools of Public Health in the European Region
WHO: World Health Organization
