# Supplementary material for: A systematic scoping review of digital health technologies during COVID-19: a new normal in primary health care delivery
Source: Health Technol (Berl). 2023 Jan 6;13(2):273–84. doi: 10.1007/s12553-023-00725-7 (PMC9816012; doi:10.1007/s12553-023-00725-7)
Supplement: Supplementary file 1 — Supplementary Material 1 [file 12553_2023_725_MOESM1_ESM.docx]

**Table S1. Search Strategy in Databases: PubMed, Scopus, and Google Scholar**

| **Database** | **Search Query** |
| --- | --- |
| MedLine via PubMed | Search: (("primary health care"[MeSH Terms]) AND ((("digital health"[Title/Abstract]) OR ("heath technology"[Title/Abstract])) OR ("healthcare technolog*"[Title/Abstract]))) AND ((((COVID-19[Title/Abstract]) OR (Sars-Cov-2[Title/Abstract])) OR ("health emergency"[Title/Abstract])) OR ((pandemic[Title/Abstract]) OR (outbreak[Title/Abstract]))) Filters: Full text, English, from 2019-2021 |
| Scopus | ( TITLE-ABS-KEY ( {primary health care} )  AND  TITLE-ABS-KEY ( "digital health"  OR  "heath technolog*"  OR  "healthcare technolog*" )  AND  TITLE-ABS-KEY ( covid-19  OR  sars-cov-2  OR  "health emergenc*"  OR  pandemic*  OR  oubreak ) )  AND  ( LIMIT-TO ( PUBYEAR ,  2021 )  OR  LIMIT-TO ( PUBYEAR ,  2020 ) )  AND  ( LIMIT-TO ( LANGUAGE ,  "English" ) ) |
| Google Scholar | “primary health care” AND ( “digital health” OR “heath technolog*” OR “healthcare technolog*” ) AND ( covid-19 OR sars-cov-2 OR “health emergenc*” OR pandemic* OR oubreak ) ); filters: aricles, custom range 2019-2021 |
